# Late respiratory alkalosis during home mechanical ventilation in amyotrophic lateral sclerosis

**DOI:** 10.1016/j.rmcr.2023.101828

**Published:** 2023-03-06

**Authors:** M.M. García, J.M. Díaz, A. Antón

**Affiliations:** a Hospital Universitario Torrecárdenas (Almería), Spain; b Servicio de Neumología del Hospital Universitario de Getafe (HUG), Madrid, Spain; c Servicio de Neumología, Hospital de la Santa Creu i Sant Pau, Barcelona, Spain

## Abstract

This demonstrative case report shows how changes in the patient's ventilatory pattern can radically modify the results of home noninvasive mechanical ventilation, and can even generate complications associated with noninvasive ventilation such as ventilatory alkalosis

Amyotrophic lateral sclerosis (ALS) is a neurologic syndrome that is characterized by the degeneration of brain and spinal cord motor neurons leading to progressive muscle weakness [1]. Respiratory failure usually occurs during the natural course of ALS, requiring treatment with noninvasive ventilation (NIV) [2].

We report the case of a 42-year-old woman diagnosed with spinal-onset ALS. The initial presentation consisted of muscle weakness of the lower extremities. Fourteen months after the onset of symptoms, she started home NIV both for respiratory failure symptoms and severe abnormalities in pulmonary function tests. A spontaneous mode with back-up respiratory rate (S/T mode) was selected, using a Stellar™ 150 (ResMed) ventilator and a full face mask (AirFit™ F20 ResMed). Ventilatory parameters are shown in Table 1. In later review visits, 4 and 6 months after starting home mechanical ventilation, the patient had increased the time of use to 24 h. Subsequent transcutaneous capnographies with NIV performed at these review visits showed normal values. Seven months after starting home mechanical ventilation, the patient developed discomfort despite the use of NIV, along with upper and lower limb paraesthesia. Arterial blood gas on NIV showed pH 7.58, PaCO_2_ 19.8 mmHg, PaO_2_ 122 mmHg, and HCO_3_ 18.9 meq/L.

**Table 1:** NIV parameters, data from built-in software and PaCO_2_ monitoring after starting mechanical home ventilation.

	4th month	7th month	8th month
NIV parameters			
IPAP cm H2O	9	9	9
EPAP cm H2O	4	4	4
Back-up respiratory rate (rpm)	16	16	10
Timin (s)	0.5	0.5	0.5
Timax (s)	1.5	1.5	1.5
Built-in software			
Mean use (hours/day)	20	24	24
Spontaneous inspiratory cycles (%)	63	22	11
Median respiratory rate (rpm)	21	16	10
Inspiratory time (s)	0.80	0.95	1
Minute ventilation (l/min)	5.5	6.9	5
PaCO_2_ (mmHg)	37	19.8	35

IPAP: inspiratory positive airway pressureEPAP: expiratory positive airway pressureTimin: minimum inspiratory timeTimax: maximum inspiratory time.

Built-in software data obtained with Rescan (Res Med®, BellaVista, NSW, Australia) are shown in Table 1. There was a progressive decrease in the spontaneous respiratory rate and an increase in controlled cycles, leading to an increase in the length of inspiratory time and overall to an increased minute ventilation, with a subsequent respiratory alkalosis secondary to hypocapnia. In Fig. 1 we can see the changes in respiratory pattern in spontaneous and controlled (“captured”) ventilation, with an increase in inspiratory time. The respiratory alkalosis was resolved by reducing the back-up respiratory rate.

**Fig. 1:**
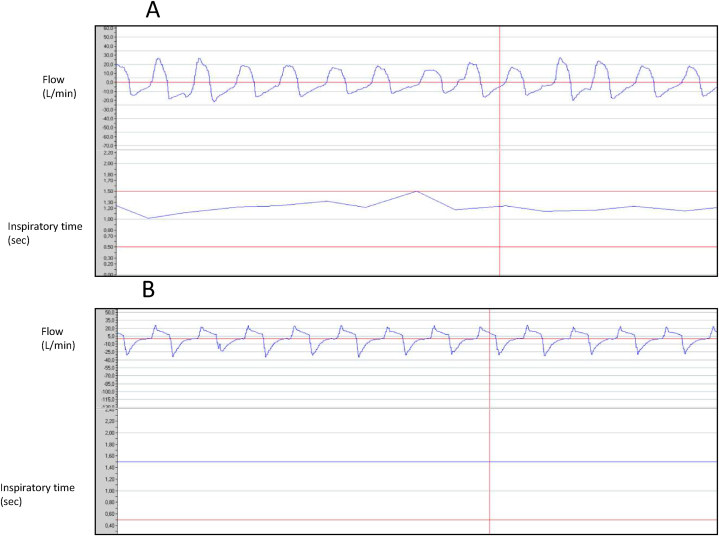
Change in ventilatory pattern over time. Legend: Flow and inspiratory time tracings at the beginning of treatment (A), and at 7 months (B). Note that at the beginning of treatment (A), with spontaneous ventilation, the inspiratory time varies cycle by cycle; at 7 months (B), with controlled ventilation cycling, the inspiratory time is fixed and prolonged.

There are five determinants of minute ventilation during NIV: ventilatory mechanics (resistance, compliance and dead space), inspiratory effort, respiratory rate, inspiratory time and the machine-delivered pressure [3,4]. In the case presented here, the machine-delivered pressure had not been modified and it seemed unlikely that the ventilatory mechanics of the patient had changed. Therefore, the increase in minute ventilation and the subsequent hypocapnia were thought to be due to modifications in the patient's own ventilatory pattern as mentioned above.

Regarding the ventilatory modality selected in our patient, pressure is the independent variable and volume is the dependent variable, which can be modified in several circumstances [5]. In the case presented here, the excessively low cycling criteria during controlled cycles increased the inspiratory time, generating a significant increase in minute ventilation. Probably, the use of new ventilation modalities in which the independent variable is ventilation and the dependent variable is pressure could have avoided the problem [6].

The development of hypocapnia and respiratory alkalosis is unusual in the natural course of a patient treated with home mechanical ventilation, and we are not aware of similar publications. Clinical guidelines do not strictly recommend arterial blood gas monitoring once home mechanical ventilation is initiated, especially when the patient uses it 24 h a day [7,8]. Nevertheless we share other authors’ opinions [9,10] about monitoring PaCO_2_ in patients treated with 24 h NIV in an attempt to ensure earlier detection of these changes.

## Declaration of competing interest

Antonio Antón declares having been part of the Res Med advisory board in Spain and received grants in 2015. No other conflicts of interest are declared.

The study was carried out entirely at the Hospital de la Santa Creu I Sant Pau, Barcelona, Spain.
